# Usefulness of Multidetector Row Computed Tomography for Predicting Cardiac Events in Asymptomatic Chronic Kidney Disease Patients at the Initiation of Renal Replacement Therapy

**DOI:** 10.1155/2013/916354

**Published:** 2013-12-02

**Authors:** Jung Eun Lee, Yong Kyu Lee, Eun Jeong Choi, Ji Sun Nam, Byoung Wook Choi, Beom Seok Kim

**Affiliations:** ^1^Department of Internal Medicine, Yonsei University, College of Medicine, Seoul 120-752, Republic of Korea; ^2^Nephrology Division, Department of Internal Medicine, NHIC, Ilsan Hospital, Goyang 410-719, Republic of Korea; ^3^Department of Radiology, Yonsei University, College of Medicine, Seoul 120-752, Republic of Korea; ^4^The Research Institute for Transplantation, Yonsei University, College of Medicine, Seoul 120-752, Republic of Korea

## Abstract

*Background*. The prevalence of coronary artery stenosis (CAS) at the initiation of renal replacement therapy (RRT) in chronic kidney disease (CKD) patients has not been fully elucidated. Although coronary angiography is the gold standard in diagnosing CAS its invasiveness and economic burden lead to searching for a noninvasive alternative method. In this study, we evaluated the prevalence of CAS by multidetector row computed tomography (MDCT) and related risk factor to articulate the usefulness of MDCT. *Method*. Seventy-four asymptomatic CKD patients who began dialysis were evaluated with echocardiography and MDCT. The patients were stratified into two groups according to CAS and coronary artery calcification score (CACS) by MDCT to detect silent CAS and evaluate its predictability for cardiac events. *Results*. CAS was seen in 24 (32.4%) of 74 asymptomatic CKD patients on MDCT. Both groups showed increasing frequencies of CAS with age (*P* < 0.01), presence of diabetes (*P* < 0.05), uric acid level (*P* < 0.01), and calcium score (*P* < 0.01). Multiple regression analysis revealed that age and uric acid level were independent risk factors for CAS and high CACS in asymptomatic CKD patients at the initiation of dialysis. Patients with both CAS and high CACS were presented with higher cardiac events rates compared to those without any of them. In Cox regression model, age and the presence of CAS and high CACS on MDCT were an independent risk factor for cardiac events in these patients. *Conclusion*. We showed that CAS was highly seen in asymptomatic CKD patients starting dialysis. Moreover, both high CACS and CAS on MDCT might predict cardiac events in these patients and MDCT can be a useful screening tool for evaluating coronary artery disease and predicting cardiovascular mortality noninvasively.

## 1. Introduction

Cardiovascular disease is the most common cause of death in patients with end-stage renal disease (ESRD) [1]. Coronary artery calcification is observed in chronic kidney disease (CKD) patients without undergoing dialysis and is progressed throughout dialysis [2, 3]. Consequently, stage 5 CKD patients at the initiation of renal replacement therapy (RRT) may already be a high-risk group for coronary artery disease (CAD) and the cardiac risk of these patients needs to be assessed accordingly. Despite a couple of reports on the high prevalence of coronary artery stenosis (CAS) in such patients with coronary angiography [4, 5], it is not generally accepted to perform invasive diagnostic procedure routinely in asymptomatic patients. Evaluation of CAD in CKD patients has been generally taken by noninvasive imaging techniques such as stress echocardiography [6] and single-photon emission computed tomography (SPECT) [7]. However, these examinations have certain limitations including high false-positive rate, misinterpretation of a hidden myocardial ischemia and/or infarction, and high prevalence of electrolyte disorders. Accordingly, more noninvasive and available tools are introduced to evaluate CAD in CKD patients and new methods such as electron beam computed tomography (EBCT) and multidetector computed tomography (MDCT) are being raised as a modality for noninvasive coronary imaging. However, EBCT is not widely used due to its costs and MDCT is more widely accepted due to its high accuracy. The purpose of this study is to evaluate the prevalence of CAS by MDCT and the usefulness of MDCT in predicting cardiac disease in asymptomatic stage 5 CKD patients at initiation of RRT.

## 2. Materials and Methods

### 2.1. Subjects and Study Design

We enrolled 74 consecutive patients (46 male, 28 female; mean age 56.5 ± 13.8 years; range 24–80 years) who started RRT in the dialysis unit of Severance Hospital, College of Medicine. Exclusion criteria included a history of angina or acute myocardial infarction, a history of malignancy, previous allergic reactions to iodine contrast media, previous percutaneous transluminal coronary stent placement, and previous bypass surgery. The study protocol was approved by the Institutional Review Board of Yonsei University Hospital, and all patients gave written informed consent.

Medical charts were reviewed for clinical history and medications. MDCT and echocardiography were performed on all patients within 7 days after the initiation of RRT, and coronary angiography was recommended for those who showed luminal diameter narrowing of >50% of any major coronary arteries. Fasting blood samples were analyzed for total cholesterol, high-density lipoprotein (HDL) cholesterol, low-density lipoprotein (LDL) cholesterol, triglycerides, plasma homocysteine, plasma fibrinogen, free fatty acid, high sensitivity C-reactive protein (hsCRP), and lipoprotein (a).

Cardiac events were defined as nonfatal angina pectoris or myocardial infarction, heart failure requiring hospitalization, and cardiac death. Unstable angina and acute myocardial infarction were diagnosed by the presence of typical angina symptoms, ischemic change or QRS change on ECG, and elevated serum cardiac enzyme levels. Heart failure was diagnosed with typical symptoms such as dyspnea, shortness of breath, or raised jugular venous pressure with systolic or diastolic dysfunction by echocardiography which is in accordance with guideline from European Society of Cardiology [8]. Cerebrovascular disease (transient ischemic attack, cerebral infarction, or cerebral hemorrhage) was confirmed both by typical symptoms with physical findings and by computed tomography (CT) or magnetic resonance imaging (MRI). Peripheral vascular disease was diagnosed when the patient had typical symptoms, abnormal ankle brachial pressure index (ABPI), positive CT or CT angiography findings, or peripheral artery stenosis confirmed by catheterization. Cardiac death was defined as death with documentation of a significant arrhythmia, cardiac arrest, or death attributable to congestive heart failure or myocardial infarction and sudden death. Elective follow-up revascularization procedures were not considered to be cardiac events.

### 2.2. MDCT Protocol

All computed tomography angiographies (CTAs) were taken by 64-MDCT (Somatom Sensation 64, Siemens, Germany). Heart rate of each patient was checked before CT scan and in patients with more than 65 beat per minute (bpm), 100 mg of Metoprolol (Selokeen, AstraZeneca Pharmaceutics, UK) was administered. After 1-hour administration of Metoprolol, heart rate was rechecked. Images for coronary calcium score were taken before contrast media injection, and scan range was from tracheobronchial bifurcation to diaphragm. Parameters of coronary calcium scan were 120 Kvp, 33 mAs, slice thickness 0.6 mm, and feed 18 mm. The protocol of CTA had scan parameters as follows: 120 Kvp, 500 mA, pitch 0.2, slice thickness 0.75 mm, collimation 0.6 mm, and gantry rotation time 0.33 sec. All CTAs were taken with a single-breath hold (10–13 s), 20 G needle through right antecubital or right wrist venous route, two kinds of nonionic water-soluble tri-iodinated contrast media (Ultravist 300 mg I/mL, Shering, Germany, and Omnipaque 300 mg I/mL, Amersham Health, Norway), and test bolus method. At first, test bolus images were taken using contrast media 10 cc with 4 cc/sec injection flow rate. Housefield unit (HU) of ascending aorta was analyzed by time-density calculation program (DynEva, Siemens, Germany). The amount of contrast media and normal saline was calculated according to cardiac scan time of each CTA with injection flow rate 4 cc/sec (contrast media) and 5 cc/sec (normal saline).

Every coronary artery was analyzed by two methods, segment and diameter. Segmental analysis was according to the 15-segment American Heart Association model of the coronary tree. In addition to the segmental analysis, if the artery showed diameter less than 1.5 mm, it was grouped as difficult to assessment.

Coronary calcium score and coronary stenosis were analyzed in all CTA. Calcium score was estimated using CAC-analysis software (Cascore, Siemens, Germany) by the Agatston score system [9] with minimal level of 130 HU and every lesion was sorted out according to its score as up to 100, 101 to 400, 401 to 999, and 1000 or more.

Degree of coronary stenosis was divided as insignificant or significant stenosis. Criterion for significant luminal narrowing was defined as over 50% of luminal narrowing by diameter compared to those of normal portions of stenotic coronary arteries, and criteria of normal portions were proximal or distal part of stenotic lesions. All radiologic studies were interpreted independently by 2 experienced radiologists without knowledge of alternate result or clinical parameter.

### 2.3. Statistical Analysis

All values are expressed as the means ± standard deviation (SD). To compare differences between the significant coronary stenosis group and nonsignificant stenosis group, independent *t*-test was applied. Multiple linear regression analysis was used to evaluate independent predictors of coronary artery stenosis (CAS) and coronary artery calcification score (CACS). To evaluate independent predictors for adverse cardiac outcomes, multivariate Cox proportional hazard model was used. Hazard ratios (HR) are presented with 95% confidence intervals in parentheses. Values of *P* < 0.05 were considered to indicate statistical significance. Statistical analysis was performed using SPSS for Windows Ver. 19.0 (SPSS, Inc., Chicago, IL, USA).

## 3. Results

### 3.1. Baseline Characteristics and Biochemical Data

The baseline characteristics of patients in present study are summarized in Table 1. Sixty-two percent of the study subjects were males. Their ages ranged from 24 to 80 years (mean age 56.5 ± 13.8 years). Thirty-eight (51.4%) patients had diabetes. Thirty-eight patients (51.4%) were started on HD, and 36 patients (48.6%) received CAPD. Twenty patients (27.0%) were smokers. Sixty-two (83.8%) patients received angiotensin-converting enzyme inhibitors or angiotensin-II receptor blockers. Thirty-three (44.6%) patients received statin treatment and 25 (33.8%) patients took calcium-based phosphate-binding agents. The mean CACS was 144.1 ± 286.5 mm^3^, ranging from 0 to 1644 mm^3^. Twenty-nine patients of 74 patients (39.1%) had no calcium deposition in their coronary arteries.

Patients were stratified into two groups according to CACS and CAS, no CACS and no CAS (CACS < 21.4 and CAS < 50%) versus CACS or CAS (≥21.4 or CAS ≥ 50%). The group with CACS or CAS showed statistically higher age (*P* < 0.01), the prevalence of diabetes (*P* < 0.05), and serum uric acid level (*P* < 0.01). No significant differences were observed in any other variables across the two groups (Table 2). A total of 24 of the 74 patients (32.4%) showed coronary artery stenosis. Seventeen of the 24 patients (70.8%) with CAS had diabetes. In the group with diabetes, the prevalence of CAS was 44.7%, while that in the group without diabetes was 19.4%. The prevalence of CAS in patients with diabetes was significantly higher than that in patients without diabetes (*P* < 0.05). Thirteen out of the 24 patients (54.1%) with severe stenosis of coronary artery (>50%) agreed to undergo coronary angiography. Eleven out of thirteen patients were the patients with diabetes and two patients were the patients without diabetes. Of these 13 patients, 6 (46.2%) had single-vessel disease, two (15.4%) had two-vessel disease, and five (38.4%) had triple-vessel disease on MDCT (Table 3).

### 3.2. Independent Predictors of Coronary Artery Stenosis and Coronary Artery Calcium Score in Asymptomatic Patients Starting Dialysis

In univariate analysis, age (hazard ratio (HR) 1.092; 95% confidence interval CI 1.043–1.144; *P* < 0.01), the presence of diabetes (HR 0.330; 95% CI 0.128–0.854; *P* < 0.05), CACS (HR 1.281; 95% CI 1.085–1.514; *P* < 0.01), uric acid levels (HR 2.090; 95% CI 1.359–3.215; *P* < 0.01), and LDL (*β* = 0.259, *P* < 0.05) were associated with CACS and CAS. Among these variables, age (*β* = 0.483, *P* < 0.01) and uric acid levels (*β* = 0.357, *P* < 0.01) were independently associated with CACS and CAS in a multivariate linear regression model (Table 4).

### 3.3. Prediction of Cardiac Events

In the univariate Cox analysis, age, the presence of diabetes, serum uric acid levels, serum LDL level, and the presence of CAS and CACS predicted cardiac events. Age (HR 1.064; 95% CI 1.018–1.112; *P* < 0.01) and the presence of CAS and CACS (HR 0.216; 95% CI 0.051–0.916) were independent risk factors in the multivariate Cox analysis (Table 5).

## 4. Discussion

Due to several limitation of coronary angiography (CAG), there has been a constant effort to replace coronary angiography with noninvasive apparatus. Single-photon emission computed tomography (SPECT) is a noninvasive pharmacologic stress test which is useful in debilitated patients, such as dialysis patients [10]. However, SPECT overlooks patients with single vessel disease, balanced multivessel disease with global ischemia, and collaterals that prevent detection of different flow [11]. Electron beam computed tomography (EBCT) was considered as a potential screening method by several groups [12, 13]. But, due to its slow scanning rate, EBCT has led to frequent artifact and low resolution, which led to inaccurate assessment of CAS [14]. Recently, 64-channel MDCT has emerged as a strong potential screening method. MDCT is superior to other apparatus, because MDCT shows reduced cardiac motion artifact and higher resolution and needs less amount of dye infusion which is crucial in CKD patients.

In this study, we have demonstrated that (1) coronary artery stenosis (CAS) is highly prevalent in asymptomatic CKD patients at the start of renal replacement therapy, (2) age and serum uric acid level are independent risk factors for CAS and CACS in MDCT, and (3) CACS and CAS on MDCT are useful in predicting cardiac events in these patients.

Generally, the prevalence of significant CAS (10%) among dialysis patients was believed not to be different from that in the general population (5~10%) [8, 15]. However, many other recent reports have contradicted this theory and have shown a high prevalence of CAS in patients who were receiving dialysis treatment [16]. Additionally, there have been a few reports about the prevalence of CAS at the initiation of dialysis therapy [4, 5, 16]. Ohtake et al. reported that 53.3% patients of asymptomatic CKD patients who underwent coronary angiography showed significant CAS [5]. In this study, the prevalence of CAS was as high as 32% but relatively low compared to a previous study by Ohtake et al. The discrepancy might be due to several reasons. Selection bias from a small sample size and a difference in ethnicity may have been attributed to such a difference. And MDCT is a less sensitive method compared to coronary angiography. However the strict exclusion criteria should have excluded possible subclinical ischemic heart disease.

Interestingly, CAS was not affected by serum Ca, P, and PTH levels which have been believed to have grave influences on calcification pathophysiology in ESRD patients. Thus, we can assume that unknown underlying risks other than Ca or P levels seem to be responsible, and certain inflammatory process may be involved. Further research is needed regarding this aspect. Our study also shows that conventional risk factors for atherosclerosis such as hypertension or hyperlipidemia did not differ between the two groups. As seen in several recent studies, our study shows that coronary artery calcification in ESRD patients can be affected by uric acid level. Elevated serum uric acid level can result in coronary calcification and eventually coronary artery stenosis through inflammatory activation, metabolic disorders, and calcium deposition [17–21].

The patients with both CACS and CAS were at higher risk for cardiovascular complication compared to those without any of them. Thus, MDCT, which can assess both parameters simultaneous, is a very useful, noninvasive tool in predicting cardiac disease in CKD patients.

However, this study has several limitations. First, this study is a single-center study with a small number of patients. Larger multicenter studies will be essential in examining MDCT for the diagnosis of coronary artery stenosis. Second, no head-to-head comparison was done with functional tests such as SPECT. Third, coronary angiography was not performed on patients whose MDCT results were negative because it was considered unethical. Therefore false-negative MDCT results were not evaluated.

In conclusion, nearly 30% of asymptomatic ESRD patients who start RRT have significant CAS. Thus, MDCT of the coronary arteries can be a useful screening method in predicting cardiovascular events in ESRD patients.

## Figures and Tables

**Table 1:** Baseline characteristics and biochemical data.

	All patients (*n* = 74)
Age (years)	56.5 ± 13.8 (24–80)
Sex (male : female)	46 : 28
Diabetes mellitus, *n* (%)	38 (51.4)
SBP (mmHg)	140.6 ± 19.5 (100–195)
DBP (mmHg)	78.1 ± 12.0 (50–120)

Hb (g/dL)	8.6 ± 1.5 (4.0–13.1)
Calcium (mg/dL)	8.3 ± 1.1 (5.5–12.3)
Phosphate (mg/dL)	5.3 ± 1.5 (1.8–11.2)
iPTH (pg/mL)	163.2 ± 124.4 (5.7–534.9)
hsCRP (mg/L)	10.1 ± 19.0 (0.15–130.0)
Albumin (g/dL)	3.4 ± 0.6 (2.2–4.6)
Total cholesterol (mg/dL)	174.3 ± 48.6 (77–439)
HDL-C (mg/dL)	39.2 ± 10.7 (20.0–71.0)
LDL-C (mg/dL)	109.6 ± 43.7 (35.8–314.8)
Triglyceride (mg/dL)	130.2 ± 55.7 (39–351)
Free fatty acid (uEq/L)	297.2 ± 199.3 (22–971)
Lipoprotein(a) (mg/dL)	51.4 ± 50.6 (0.7–299)
Fibrinogen (mg/dL)	474.3 ± 133.8 (264–958)
PAI-1	16.7 ± 10.0 (6.7–51.3)
Homocysteine (umol/L)	20.5 ± 14.5 (5.4–124.4)

EF (%)	61.3 ± 10.0 (28–78)
LVMI	35.5 ± 13.5 (13.7–78.1)

CACS	144.1 ± 286.5 (0–1644)

Cause of ESRD, *n* (%)	
DM nephropathy	41 (55.4%)
Hypertensive nephropathy	19 (25.6%)
Chronic Glomerulonephritis	8 (10.8%)
Etc	4
unknown	2

All values are expressed as mean ± SD; SBP: systolic blood pressure; DBP: diastolic blood pressure; Hb: hemoglobin; iPTH: intact parathyroid intact hormone; hsCRP: high sensitivity C-reactive protein; HDL: high density lipoprotein; LDL: low density lipoprotein; PAI-1: plasminogen activator inhibitor-1; EF: ejection fraction; LVMI: left ventricular mass index; CACS: coronary artery calcium score.

**Table 2:** Basic characteristics of the two groups according to CACS and CAS by MDCT.

	No CACS/CAS (*n* = 33)	CACS or CAS (*n* = 41)	*P* value
Age (years)	49.0 ± 14.2	62.5 ± 10.2	<0.01
Sex (male : female)	20 : 13	26 : 15	NS
Diabetes mellitus, *n* (%)	12 (36.3%)	26 (63.4%)	<0.05
SBP (mmHg)	140.3 ± 21.9	140.8 ± 17.6	NS
DBP (mmHg)	79.7 ± 12.8	76.6 ± 11.4	NS

Hb (g/dL)	8.5 ± 1.8	8.8 ± 1.3	NS
Calcium (mg/dL)	8.2 ± 1.1	8.3 ± 1.2	NS
Phosphate (mg/dL)	5.6 ± 1.8	5.0 ± 1.2	NS
iPTH (pg/mL)	184.0 ± 137.9	145.9 ± 110.9	NS
Uric acid	6.2 ± 1.4	7.5 ± 1.2	<0.01
hsCRP (mg/L)	10.5 ± 25.0	9.6 ± 13.4	NS
Albumin (g/dL)	3.4 ± 0.7	3.4 ± 0.5	NS
TC (mg/dL)	167.5 ± 34.9	179.7 ± 57.0	NS
HDL-C (mg/dL)	42.0 ± 12.3	37.2 ± 8.9	NS
LDL-C (mg/dL)	99.0 ± 31.6	117.4 ± 49.6	NS
Triglyceride (mg/dL)	124.3 ± 48.7	134.8 ± 60.9	NS
Free fatty acid (uEq/L)	299.3 ± 234.5	295.5 ± 169.9	NS
Lipoprotein(a) (mg/dL)	55.4 ± 47.3	48.3 ± 53.3	NS
Fibrinogen (mg/dL)	481.7 ± 130.5	468.4 ± 138.0	NS
PAI-1	15.8 ± 7.6	17.2 ± 11.4	NS
Homocysteine (umol/L)	22.1 ± 20.9	19.2 ± 6.6	NS

EF (%)	61.3 ± 12.7	61.4 ± 7.8	NS
LVMI	33.1 ± 9.0	36.9 ± 15.7	NS

All values are expressed as mean ± SD; SBP: systolic blood pressure; DBP: diastolic blood pressure; Hb: hemoglobin; iPTH: intact parathyroid intact hormone; hsCRP: high sensitivity C-reactive protein; HDL: high density lipoprotein; LDL: low density lipoprotein; PAI-1: plasminogen activator inhibitor-1; EF: ejection fraction; LVMI: left ventricular mass index; CACS: coronary artery calcium score; NS: not significant.

**Table 3:** Prevalence of CAS by MDCT among 74 asymptomatic CKD patients.

		DM (*n* = 38)	Non-DM (*n* = 36)	PCI (*n* = 13)
CAS positive, *n* (%)	24 (32.1)	17	7	13
One vessel, *n* (%)	15 (62.5)	9	6	6
Two vessels, *n* (%)	3 (12.5)	1	2	2
Three vessels, *n* (%)	6 (25.0)	5	1	5

**Table 4:** Multiple linear regressions of factors associated with CAS and CACS in CKD patients at the start of dialysis.

Variables	Univariate analysis		Multivariate analysis	
	*β*	*P* value	*β*	*P* value
Age	0.488	<0.01	0.401	<0.01
diabetes	0.269	<0.05	0.153	NS
CACS	0.452	<0.01	0.152	NS
Uric acid	0.436	<0.01	3.071	<0.05
LDL	0.209	NS	—	—
EF	0.007	NS	—	—
SBP	0.012	NS	—	—
hsCRP	0.025	NS	—	—

CACS: coronary artery calcium score; LDL: low density lipoprotein; EF: ejection fraction; SBP: systolic blood pressure; hsCRP: high sensitivity C-reactive protein; NS: not significant.

**Table 5:** Cox regression models in CKD patients at the start of dialysis.

	HR (95% CI)	*P* value
Age	1.064 (1.018~1.112)	<0.01
The presence of diabetes	0.469 (0.184~1.196)	NS
SBP	1.009 (0.981~1.038)	NS
Uric acid	0.726 (0.515~1.023)	NS
LDL	0.987 (0.972~1.001)	NS
CACS + CAS	0.216 (0.051~0.916)	<0.05

SBP: systolic blood pressure; LDL: low density lipoprotein; CACS: coronary artery calcium score; CAS: coronary artery stenosis; NS: not significant.
